# *UGT1A1* Polymorphism for Irinotecan Dose Escalation in Patients with *BRAF*-Mutated Metastatic Colorectal Cancer Treated with First-Line Bevacizumab and FOLFIRI

**DOI:** 10.1155/2021/6686517

**Published:** 2021-03-09

**Authors:** Yi-Chien Hsieh, Tsung-Kun Chang, Wei-Chih Su, Ching-Wen Huang, Hsiang-Lin Tsai, Yen-Cheng Chen, Ching-Chun Li, Po-Jung Chen, Tzu-Chieh Yin, Cheng-Jen Ma, Jaw-Yuan Wang

**Affiliations:** ^1^Division of Colorectal Surgery, Department of Surgery, Kaohsiung Medical University Hospital, Kaohsiung Medical University, Kaohsiung, Taiwan; ^2^Graduate Institute of Clinical Medicine, College of Medicine, Kaohsiung Medical University, Kaohsiung, Taiwan; ^3^Department of Surgery, Faculty of Medicine, College of Medicine, Kaohsiung Medical University, Kaohsiung, Taiwan; ^4^Division of Colorectal Surgery, Department of Surgery, Kaohsiung Municipal Hsiaokang Hospital, Kaohsiung, Taiwan; ^5^Department of Surgery, Kaohsiung Municipal Tatung Hospital, Kaohsiung Medical University, Kaohsiung, Taiwan; ^6^Division of Digestive and General Surgery, Department of Surgery, Kaohsiung Medical University Hospital, Kaohsiung Medical University, Kaohsiung, Taiwan; ^7^Graduate Institute of Medicine, College of Medicine, Kaohsiung Medical University, Kaohsiung, Taiwan; ^8^Center for Cancer Research, Kaohsiung Medical University, Kaohsiung, Taiwan; ^9^Cohort Research Center, Kaohsiung Medical University, Kaohsiung, Taiwan

## Abstract

**Background:**

Patients with metastatic colorectal cancer (mCRC) and *BRAF* V600E mutation have a poor prognosis, with a median progression-free survival (PFS) of only 5–7 months after initial therapy. The current standard first-line chemotherapy for these patients includes FOLFOX or FOLFIRI plus bevacizumab. In this study, we explored the effects and oncological outcomes of *UGT1A1* polymorphism for irinotecan escalation in patients with *BRAF-*mutated mCRC. *Patients and Methods*. This retrospective study included 17 patients with *BRAF-*mutated mCRC between April 2016 and December 2019. *UGT1A1* genotyping was performed on all patients prior to initiating bevacizumab plus FOLFIRI chemotherapy. The primary endpoint was PFS, and the secondary endpoints were toxicity, response rate, disease control rate, and overall survival (OS).

**Results:**

Fifteen and two patients had *UGT1A1* 1∗/1∗ and 1∗/28∗, respectively. Eight underwent irinotecan dose escalation with tolerable adverse effects (AEs), and nine maintained an irinotecan dose of 180 mg/m^2^ or required deescalation to 150 mg/m^2^ due to intolerable AEs. After a median follow-up period of 15.7 (range, 3–54) months, the median PFS and OS were 9.4 and 15.7 months, respectively. Grade 3/4 AEs were observed in three (6%) patients. The disease control and partial response rates were 64.7% and 11.8%, respectively, indicating that most patients (14, 82.3%) could maintain this as a first-line line therapy with stable disease or proceed to second-line therapy if disease progression occurred, thereby maintaining acceptable performance status.

**Conclusions:**

The oncological outcomes of patients with *BRAF-*mutated mCRC treated using FOLFIRI plus bevacizumab with irinotecan dose escalation as a first-line therapy are acceptable with tolerable AEs; this may be a feasible treatment option in such patients. Pretherapeutic *UGT1A1* genotyping-guided dose adjustment can achieve favorable outcomes.

## 1. Introduction

Colorectal cancer (CRC) is the third most common malignancy and cause of cancer-related deaths worldwide, with approximately 25% of patients with CRC presenting with metastatic lesions at initial diagnosis [1–3]. Despite advances in systemic chemotherapy, target therapy, and surgical techniques, the median overall survival (OS) of patients with metastatic CRC (mCRC) is approximately 30 months [4, 5]. Studies have increasingly focused on the molecular pathology and genetic aberrations involved in CRC to stratify patients for individualized treatments [6]. For instance, patients with CRC who have *RAS* mutations are excluded from treatments using antiepidermal growth factor receptor (EGFR) monoclonal antibody [7, 8]. Among these new findings, *BRAF* proto-oncogene mutation, a marker of poor prognosis, was observed in 8%–15% of patients with mCRC exhibiting aggressive tumor biology and poor response to standard therapy [1, 2, 6, 9, 10].

The *BRAF* oncogene encodes a serine/threonine kinase downstream of *RAS* in the MAPK pathway, playing a key role in the regulation of cellular proliferation [11]. Most *BRAF* mutations are found in a single amino acid substitution in codon 600 of exon 15 (V600E), and they are associated with unique clinical characteristics, including female sex, older age, right-sided tumor, and peritoneal and distant lymph node metastasis [6, 12, 13].

The current standard first-line chemotherapy for patients with *BRAF*-mutated mCRC includes fluoropyrimidine-based doublet chemotherapy—FOLFOX or FOLFIRI—plus bevacizumab; this regimen affords a median progression-free survival (PFS) of 5–7 months [14]. The phase 3 TRIBE study revealed a trend toward improved OS in patients with *BRAF* mutation receiving triplet chemotherapy FOLFOXIRI plus bevacizumab although no statistical significance was observed [15]. Because toxicity management remains a concern, the use of FOLFOXIRI as a first-line treatment option reflects that patients with mCRC may not exhibit adequate performance status to maintain the treatment. Our previous study revealed that patients with mCRC receiving escalated doses of irinotecan based on uridine diphosphate glucuronosyltransferase 1A1 (*UGT1A1*) genotyping exhibited favorable clinical responses and outcomes [3, 16, 17]. In the present retrospective study, we used real-world data and explored the effects and oncological outcomes of FORFIRI plus bevacizumab as first-line therapy with irinotecan dose escalation according to *UGT1A1* polymorphism in patients with *BRAF*-mutated mCRC.

## 2. Material and Methods

### 2.1. Patients

This retrospective chart review identified 24 patients with histologically or radiologically proven mCRC with *BRAF* mutation between April 2016 and December 2019. All patients underwent *UGT1A1* genotyping before the bevacizumab plus FOLFIRI regimen was started [3]. The recommended starting dose of irinotecan was 180 mg/m^2^. The dose was escalated in increments of 20–30 mg/m^2^ until grade 3/4 AEs occurred, depending on the *UGT1A1* genotype. We analyzed the response rate, disease control rate (DCR), PFS, OS, and grade 3/4 AEs.

## 3. Methods

### 3.1. *BRAF* Mutation Analysis

We extracted DNA from formalin-fixed, paraffin-embedded (FFPE) CRC tissue samples for clinical *BRAF* mutation analysis by direct sequencing. The FFPE samples were deparaffinized and air-dried; subsequently, DNA was isolated using the proteinase K and QIAamp DNA Micro Kit (QIAGEN) in accordance with the manufacturer's instructions. We designed a set of primers for high-resolution melting (HRM) analysis that were specific for the *BRAF* V600E mutation while fulfilling the requirements of the LightCycler® 480 System Gene Scanning Assay. Primer3 free software was used to design the primers used in this study. The forward and reverse primer sequences were 5ʹ-CATAATGCTTGCTCTGATAGGAAA-3ʹ and 5ʹ-TCAGCACATCTCAGGGCCAAA-3ʹ, respectively. All the primers synthesized were of standard molecular biology quality (Protech Technology Enterprise Co., Ltd., Taiwan).

Polymerase chain reaction (PCR) was performed with 10 *μ*L as the final volume by using a Light Cycler® 480 High-Resolution Melting Master (Reference 04909631001, Roche Diagnostics) with 1× buffer (containing Taq polymerase, nucleotides, and ResoLight dye) and 20 ng of DNA. The primers and MgCl_2_ were used at concentrations of 0.25 *μ*M and 2.5 mM, respectively, to identify *BRAF* mutation status. The HRM analyses were performed using the LightCycler® 480 Instrument (Roche Diagnostics) provided with the software LightCycler® 480 Gene Scanning Software (version 1.5; Roche Diagnostics). The PCR program required an SYBR Green I filter (533 nm). It comprised an initial denaturation-activation step at 95°C for 10 min, followed by a 45-cycle program (denaturation at 95°C for 15 s, annealing at 60 or 62°C for 15 s, and elongation at 72°C for 15 s with fluorescence reading; acquisition mode: single). The melting program included the following three steps: denaturation at 95°C for 1 min, renaturation at 40°C for 1 min, and subsequent melting that consisted of continuous fluorescence reading from 60 to 90°C at a rate of 25 acquisitions per 1°C.

After HRM analysis, the samples were purified using a PCR-M™ clean-up system (Viogen, Sunnyvale, CA, USA). The PCR products generated after HRM were directly sequenced. The sequencing reaction was performed at a final volume of 10 *μ*L, including 1 *μ*L of the purified PCR product, 2.5 *μ*M of one of the PCR primers, 2 *μ*L of the ABI PRISM terminator cycle sequencing kit v3.1 (Applied Biosystems, Foster City, CA, USA), and 2 *μ*L of 5× sequence buffer. The sequencing program is a 25-cycle PCR program (denaturation at 96°C for 10 s, annealing at 50°C for 5 s, and elongation at 60°C for 4 min). Sequence detection was performed using the ABI Prism 3130 Genetic Analyzer (Applied Biosystems) according to standard protocols [18].

### 3.2. *UGT1A1* Mutation Analysis

Constitutional gene polymorphisms were analyzed with DNA extraction from 4 mL of peripheral blood by using the Puregene DNA Isolation Kit (Gentra Systems Inc., Minneapolis, MN, USA). The patients' genomic DNA was analyzed using direct sequencing to determine the genotype of the *UGT1A1* promoter region. The primers used in this study were designed using Primer3. The forward and reverse primer sequences were 5ʹ-AGTCACGTGACACAGTCAAACA-3ʹ and 5ʹ-CTTTGCTCCTGCCAGAGGTT-3ʹ, respectively. The PCR volume was 40 *μ*L, and the PCR conditions for glutathione S-transferase pi 1 (*GSTP1*) were as follows: 94.0°C for 5 min, 30 cycles of denaturation for 30 s at 94.0°C, annealing for 20 s at 67.5°C, primer extension for 20 s at 72.0°C, and final extension for 10 min at 72.0°C. We performed a fragment analysis of the PCR products to verify the genotypes through automated capillary electrophoresis by using the ABI PRISM 310 Genetic Analyzer (Applied Biosystems, Foster City, CA, USA), and the genotypes were analyzed using GeneScan and Genotyper (Applied Biosystems) [19].

### 3.3. Postchemotherapy Surveillance

The treatment response was assessed using computed tomography, magnetic resonance imaging, or positron emission tomography, and the best responses were recorded. The first response assessment was usually performed after the sixth cycle for patients who received bevacizumab combined with FOLFIRI. The criteria of the Response Evaluation Criteria in Solid Tumors (RECIST; version 1.1) [20] were used to classify patient responses. The AEs were monitored and graded in each cycle according to the National Cancer Institute Common Terminology Criteria for Adverse Events (version 4.3; http://ctep.cancer.gov/reporting/ctc.html). The most effective response was defined as the best result recorded by the investigators. The median follow-up period was 14.5 (range, 3–54) months. This study was approved by the Institutional Review Board of Kaohsiung Medical University Hospital (KMUHIRB-2012-03-02(II)). All patients signed an informed consent form. PFS was the primary endpoint, and the secondary endpoints were toxicity, response rate, DCR, and OS.

### 3.4. Statistical Analysis

SPSS (version 19.0; SPSS, Chicago, IL, USA) was used for all data analyses. PFS was defined as the time from the initiation of study treatment to the first radiological progression or tumor-related death, whichever came first. OS was defined as the time from initiation of study treatment to death from any cause. The Kaplan–Meier method was used to calculate PFS and OS, and a log-rank test was used to compare time-to-event distributions. Statistical significance was set to *P* < 0.05.

## 4. Results

**Figure 1:**
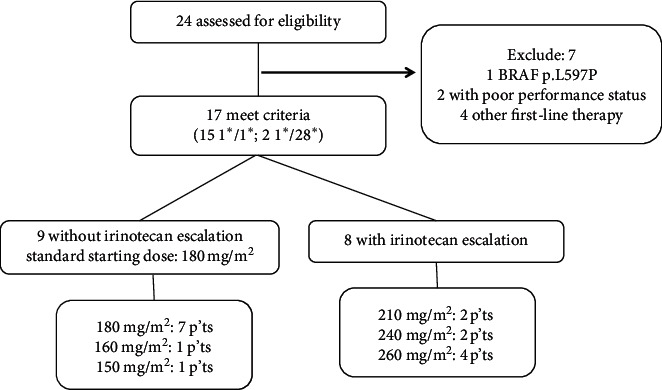
Flowchart of patient selection.

We retrospectively reviewed 24 patients with *BRAF*-mutated mCRC. Seven were excluded for the following reasons: one exhibited *BRAF* mutation at position 597 (p.L597P); two showed poor performance status (Eastern Cooperative Oncology Group performance status (ECOG) > 2), with intolerance to systemic therapy; and four received different regimens (Figure 1). The median age of the 17 included patients (8 men and 9 women) was 56 (range, 35–81) years. Nine and eight patients presented with right- and left-sided CRC, respectively. Thirteen (76.5%) of the tumors were moderately differentiated, and four (23.5%) were poorly differentiated. The most common metastatic site was the liver (12 of 17, 70.5%) followed by the peritoneum (7, 41.2%), lung (5, 29.4%), and distant lymph nodes (4, 23.5%). Six (35.3%) patients had single-site metastasis, and 11 (64.7%) had multiple metastatic sites. Fifteen (88.2%) and two (11.8%) patients had *UGT1A1* 1∗/1∗, respectively (Table 1).

Eight (47.1%) patients underwent irinotecan dose escalation with tolerable AEs, and nine (52.9%) were maintained at the recommended irinotecan dose (180 mg/m^2^) or switched to a lower dose (150 mg/m^2^) because of intolerable AEs. No significant differences were noted between the two groups (Table 2). In total, 5 (29.4%), 10 (58.8%), and 2 (11.8%) patients experienced grade 1, 2, and 3 toxicities, respectively. No grade 4/5 toxicities were reported. The most frequently reported AEs (any grade) included anemia (76.5%), fatigue (58.8%), nausea (52.9%), leukopenia (52.9%), and hair loss (47%). Grade 3 AEs were observed in two (11.8%) patients (one with grade 3 nausea/vomiting, one with grade 3 leukopenia; Table 3).

**Figure 2:**
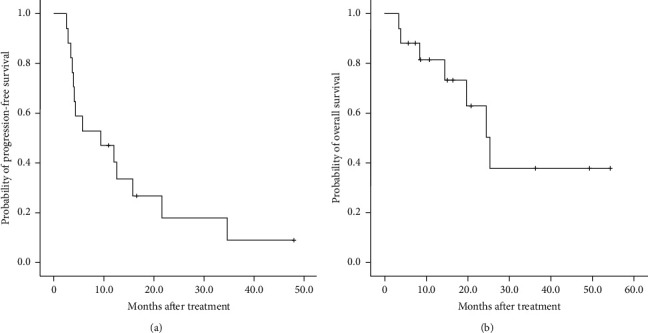
Kaplan–Meier survival curves for (a) median progress-free survival of 9.4 months and (b) median overall survival of 15.7 months for all 17 patients.

**Figure 3:**
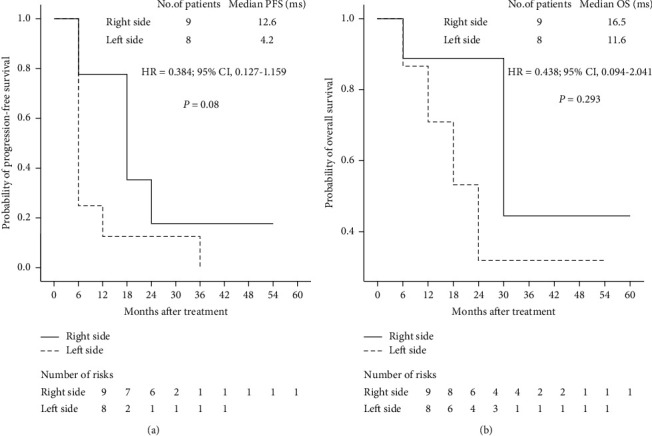
Kaplan–Meier survival curves for (a) progress-free survival and (b) overall survival stratified by tumor sidedness. A trend of favorable PFS and OS was observed in patients with right-sided mCRC.

**Figure 4:**
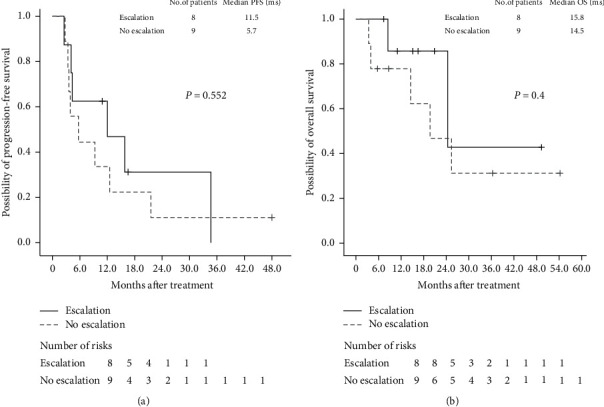
Kaplan–Meier survival curves for (a) progress-free survival and (b) overall survival stratified by irinotecan escalation or no escalation.

The DCR was 64.7% (11 of 17 patients), and the response rate was 11.8% (2 of 17, Table 1). The median PFS and OS were 9.4 and 15.7 months, respectively (Figures 2(a) and 2(b)). Regarding tumor sidedness, the median PFS was 4.2 and 12.6 months in patients with left-sided and right-sided mCRC, respectively (*P*=0.08, HR: 0.384, 95% CI: 0.127–1.159, Figure 3(a)), whereas the median OS was 11.6 and 16.5 months in patients with left-sided mCRC and those with right-sided mCRC, respectively (*P*=0.293, HR: 0.438, 95% CI: 0.094–2.041, Figure 3(b)). Although the differences were not significant, favorable PFS trends were observed in patients with right-sided mCRC. Regarding irinotecan escalation, the median PFS was 11.5 and 5.7 months in patients with and without dose escalation, respectively (*P*=0.552, 95% CI: 7.307–21.485, Figure 4(a)), whereas the median OS was 15.8 and 14.5 months in patients with and without escalation, respectively (*P*=0.40, 95% CI: 18.241–32.559, Figure 4(b)). Although no significant difference in PFS and OS was observed between the two groups, a trend of improved PFS was found in patients in the escalation group.

## 5. Discussion

Patients with *BRAF-*mutated mCRC have aggressive tumor biology and poor prognosis. Several clinical trials have been performed to improve survival and DCR [14, 21]. Here, we retrospectively reviewed the clinical characteristics, treatment regimens of FOLFIRI with irinotecan dose escalation plus bevacizumab, toxicities, and oncological outcomes of patients with *BRAF-*mutated mCRC at a single tertiary center in a real-world setting.

No significant sex, primary tumor location, or metastatic site differences were observed in our study, which was inconsistent with the results of a previous study [13]. Although some studies have reported that older age, female sex, right-sided tumor, and peritoneal metastasis are associated with an increased likelihood of *BRAF* V600E mutation in Caucasian people [13, 22–25], our study provides different data in Asian patients with mCRC. Therefore, the absence of significant differences in age, sex, metastatic site, and tumor location may be attributable to the differences in the size or ethnicity of the sample population.

The standard first-line chemotherapeutic treatment for advanced *BRAF-*mutated mCRC is a fluoropyrimidine-based cytotoxic regimen, including either irinotecan or oxaliplatin combined with bevacizumab [26]. This combination was evaluated retrospectively in 127 patients with *BRAF-*mutated mCRC; the results revealed poor PFS of 6.4 and 5.4 months with oxaliplatin- and irinotecan-based regimens plus bevacizumab as the first-line chemotherapy treatment, respectively [10, 27]. To counter the disappointing result, the TRIBE study applied a more aggressive strategy of FOLFOXIRI (5-FU, leucovorin, oxaliplatin, and irinotecan) plus bevacizumab in a small subgroup of patients with *BRAF-*mutated CRC [15, 28] and revealed the median OS and PFS to be 19.0 and 7.5 months, respectively, compared with the OS of 10.7 and 5.5 months in the FOLFIRI plus bevacizumab group, respectively, after a median follow-up of 48.1 months (HR: 0.54, 95% CI: 0.24–1.20 and HR: 0.57; 95% CI: 0.27–1.23, respectively). Furthermore, the OS and PFS were more favorable in patients with right-sided mCRC on FOLFOXIRI plus bevacizumab than in those with left-sided mCRC on FOLFIRI plus bevacizumab, although the differences were not significant. Hence, the study concluded that FOLFOXIRI plus bevacizumab may be preferred as the first-line treatment for clinically selected patients with right-sided mCRC irrespective of their *RAS* and *BRAF* mutation status [15, 28]. In our study, the median OS and PFS were 15.7 and 9.4 months, respectively, indicating favorable outcomes in patients with *BRAF-*mutated mCRC treated with irinotecan escalation strategy. Regarding tumor sidedness, although the differences were not significant, favorable PFS and OS trends were observed in patients with right-sided mCRC, similar to the TRIBE study [15].

The improvement in applying the FOLFOXIRI regimen also increases toxicity. A phase III trial randomly assigned 244 patients to receive either infusional FOLFOXIRI or infusional FOLFIRI as the first-line treatment for mCRC. The FOLFOXIRI group experienced higher grade 2-3 peripheral neurotoxicity (0% vs. 19%; *P* < 0.001) and grade 3-4 neutropenia (28% vs. 50%; *P* < 0.001) than did the FOLFIRI group [29]. In the TRIBE study, the incidence of grade 3 or 4 neutropenia, diarrhea, stomatitis, and neurotoxicity (i.e., peripheral neuropathy) was significantly higher in the FOLFOXIRI plus bevacizumab group than in the FOLFIRI plus bevacizumab group [30]. In the present study, only 2 of 17 (11.8%) patients experienced grade 3 toxicities from irinotecan escalation according to *UGT1A1* genotyping. Because of tolerable AEs, patients can proceed to second-line therapy with acceptable performance status after our first-line therapy.

Irinotecan must be converted by a carboxylesterase to SN-38, which is actively cytotoxic and detoxified by the glucuronidation activity of UGT; therefore, the *UGT1A1* genotype represents the development of drug-associated AEs. In a genotype-directed dose-determination study on irinotecan dose escalation in first-line FOLFIRI for mCRC, Marcuello et al. reported that compared with patients of other genotypes, patients with homozygous *UGT1A1*∗28/∗28 developed more severe irinotecan-associated AEs more frequently [31]. By contrast, clinical presentations in patients with *UGT1A1*∗1/∗28 exhibit individual variation; however, these patients generally tolerate the recommended initial irinotecan dose of 180 mg/m^2^ [32]. Conversely, patients with homozygous *UGT1A1*∗1/∗1 genotype are more tolerant of irinotecan-associated AEs and can tolerate an irinotecan dose as high as 260 mg/m^2^ [32]. Patients with the *UGT1A1*∗1/∗1 and ∗1/∗28 genotypes received high doses of irinotecan to achieve a favorable ORR without significant AEs in a phase II trial by Paez et al. [33]. According to the pan-Asian adapted ESMO consensus guidelines, patients with a favorable *UGT1A1* genotype (homozygous wild ∗1/∗1 and heterozygous ∗1/∗28) can tolerate high-dose irinotecan without significant toxicity [34]. Here, no significant differences in PFS and OS were observed in patients in the escalation group, but a trend of better PFS and OS was found in our study. Future studies should include a larger sample size to verify the effect of irinotecan dose escalation.

Patients with mCRC who have pretherapeutic *UGT1A1* genotyping and subsequent irinotecan dose adjustments tend to exhibit favorable responses and outcomes without a significant increase in toxicities when treated with FOLFIRI plus bevacizumab [3, 16, 17, 19]. Compared with the TRIBE study results, our data reveal acceptable OS and PFS but less severe AEs. Moreover, 82.3% of the patients with *BRAF-*mutated mCRC undergoing first-line treatment with this strategy could maintain this therapy with stable disease or proceed to second-line therapy in cases of disease progression, which means they still can preserve an acceptable performance status.

A second-line therapy using a combination of a *BRAF* inhibitor or *MEK* inhibitor with anti-EGFR may become the new standard of care for patients previously treated for *BRAF* V600E mutated CRC in cases of *BRAF*-mutated mCRC with aggressive characteristics [35]. The administration of triplet therapy led to approximately 50% grade 3 or 4 AEs; therefore, maintaining patients' performance status and encouraging them to undergo further therapy to prolong their lives is crucial [35].

The limitations of this study include its small sample size and data from a single center. Further prospective multicenter studies should be conducted to verify our results.

## 6. Conclusion

In summary, the oncological outcomes of patients with *BRAF-*mutated mCRC treated using FOLFIRI plus bevacizumab with irinotecan dose escalation as a first-line therapy are acceptable with tolerable AEs; thus, it can be a feasible treatment option in selected patients.

## Figures and Tables

**Table 1:** Baseline characteristics of 17 patients with *BRAF-*mutated mCRC stratified by tumor sidedness.

Characteristic	All patients (*N* = 17)	Group A (right side) (*N* = 9)	Group B (left side) (*N* = 8)	*P* value
*Age (years)*				
Median ± SDa (range)	58 ± 12.9 (35–81)	58 ± 10.8 (36–73)	53.5 ± 15.8 (35–81)	1.000
*Gender*				0.109
Male	8 (47.1%)	6 (66.7%)	2 (25%)	
Female	9 (52.9%)	3 (33.3%)	6 (75%)	
*Histology*				1.000
WD	0 (0%)	0 (0%)	0 (0%)	
MD	13 (76.5%)	7 (77.8%)	6 (75%)	
PD	4 (23.5%)	2 (22.2%)	2 (25%)	
*Site of metastasis*				0.580
1	6 (35.3%)	3 (33.3%)	3 (37.5%)	
2	7 (41.2%)	3 (33.3%)	4 (50.0%)	
≥3	4 (23.5%)	3 (33.3%)	1 (12.5%)	
*BMI (kg/m* ^*2*^)				0.871
Mean ± SDa	24.4 ± 2.48	23.4 ± 2.98	23.1 ± 6.44	
*UGT1A1*				0.735
∗1/∗1	15 (88.2%)	8 (88.9%)	7 (87.5%)	
∗1/∗28	2 (11.8%)	1 (11.1%)	1 (12.5%)	
*Response*				0.067
Complete response	0 (0.0%)	0 (0%)	0 (0%)	
Partial response	2 (11.8%)	0 (0%)	2 (25.0%)	
Stable disease	9 (52.9%)	7 (77.8%)	2 (25.0%)	
Progressive disease	6 (35.3%)	2 (22.2%)	4 (50.0%)	
*Responder*				0.11
Yes	2 (11.8%)	0 (0%)	2 (25%)	
No	15 (89.2%)	9 (100%)	6 (75%)	
*DCR*				0.23
Yes	11 (64.7%)	7 (77.8%)	4 (50%)	
No	6 (35.3%)	2 (22.2%)	4 (50%)	

WD: well differentiated; MD: moderately differentiated; PD: poorly differentiated; DCR: disease control rate.

**Table 2:** Baseline characteristics of 17 patients with *BRAF*-mutated mCRC divided into irinotecan escalation and no irinotecan escalation groups.

Characteristic	Group A (irinotecan escalation) (*N* = 8)	Group B (no irinotecan escalation) (*N* = 9)	*P* value
*Age (years)*			
Median ± SDa (range)	59 ± 11.9 (36–72)	51 ± 14.6 (35–81)	0.459
*Gender*			0.057
Male	6 (75%)	2 (22.2%)	
Female	2 (25%)	7 (77.8%)	
*Histology*			0.576
WD	0 (0%)	0 (0%)	
MD	7 (87.5%)	6 (66.7%)	
PD	1 (12.5%)	3 (33.3%)	
*Site of metastasis*			0.959
1	3 (37.5%)	3 (33.3%)	
2	3 (37.5%)	4 (44.5%)	
≥3	2 (25%)	2 (22.2%)	
*BMI (kg/m* ^*2*^)			0.623
Mean ± SDa	23.9 ± 3.47	22.7 ± 5.83	
*Response*			0.697
Complete response	0 (0%)	0 (0%)	
Partial response	1 (12.5%)	1 (11.1%)	
Stable disease	5 (62.5%)	4 (44.4%)	
Progressive disease	2 (25%)	4 (44.4%)	
*Responder*			1.00
Yes	1 (12.5%)	1 (11.1%)	
No	7 (87.5%)	8 (88.9%)	
*DCR*			0.62
Yes	6 (75%)	5 (55.6%)	
No	2 (25%)	4 (44.4%)	

**Table 3:** Toxicities of 17 patients with mCRC.

Adverse effect	Grades 1–2 (%)	Grade ≥ 3 (%)	Any grade (%)
Anemia	13 (76.5)	0 (0)	13 (76.5)
Fatigue	10 (58.8)	0 (0)	10 (58.8)
Nausea	8 (47.1)	1 (5.9)	9 (52.9)
Neutropenia	8 (47.1)	1 (5.9)	9 (52.9)
Hair loss	8 (47.1)	0 (0)	8 (47.1)
Thrombocytopenia	6 (35.3)	0 (0)	6 (35.3)
Abnormal liver function	6 (35.3)	0 (0)	6 (35.3)
Vomiting	3 (17.6)	1 (5.9)	4 (23.5)
Acute kidney injury	3 (17.6)	0 (0)	3 (17.6)
Diarrhea	2 (11.8)	0 (0)	2 (11.8)
Paresthesia	2 (11.8)	0 (0)	2 (11.8)

## Data Availability

The data used to support the findings of this study have been deposited in the DOI repository.
